# Drug Use in Night Owls May Increase the Risk for Mental Health Problems

**DOI:** 10.3389/fnins.2021.819566

**Published:** 2022-01-11

**Authors:** Jeevan Fernando, Jan Stochl, Karen D. Ersche

**Affiliations:** ^1^Department of Psychiatry, University of Cambridge, Cambridge, United Kingdom; ^2^Department of Kinanthropology and Humanities, Charles University, Prague, Czechia; ^3^Department of Systems Neuroscience, University Medical Center Hamburg-Eppendorf, Hamburg, Germany

**Keywords:** chronotype, anxiety, tobacco, alcohol, cannabis, mediation analysis, depression, online

## Abstract

Drugs of abuse are widely known to worsen mental health problems, but this relationship may not be a simple causational one. Whether or not a person is susceptible to the negative effects of drugs of abuse may not only be determined by their addictive properties, but also the users’ chronotype, which determines their daily activity patterns. The present study investigates the relationship between chronotype, drug use and mental health problems in a cross-sectional community sample. Participants (*n* = 209) completed a selection of questionnaires online, including the Munich Chronotype Questionnaire, the Depression Anxiety Stress Scale, the Alcohol Use Disorder Identification Test, the Cannabis Use Disorder Identification Test and the Fagerström Test for Nicotine Dependence. We conducted multiple regression models to determine relationships between participants’ chronotype and their reported mental health symptoms and then estimated mediation models to investigate the extent to which their drug consumption accounted for the identified associations. Chronotype was significantly associated with participants’ overall mental health (β = 0.16, *p* = 0.022) and their anxiety levels (β = 0.18, *p* = 0.009) but not with levels of depression or stress. However, both relationships were fully mediated by participants’ overall drug consumption. Thus, late chronotypes, so-called “night owls”, not only use more drugs but consequently have an increased risk for developing anxiety and deteriorating mental health status. This group may be particularly vulnerable to the negative psychological effects of drugs. Our results point toward the importance of considering chronotype in designing preventative and therapeutic innovations, specifically for anxiety, which at present has been largely neglected.

## Introduction

The relationship between drug use and mental health has been well established. Tobacco, alcohol and cannabis are the most widely used drugs of abuse, and each of them has been associated with mood disorders (Boden and Fergusson, 2011; Jamal et al., 2012; Lev-Ran et al., 2014; Hser et al., 2017; Mathew et al., 2017). The relationship between drug use and mental health has become even more obvious during the COVID-19 pandemic, when not only the use of cannabis and alcohol increased but also the prevalence of mental health symptoms (Vanderbruggen et al., 2020; Xiong et al., 2020). This recent increase in mental health problems warrants further investigation into the relationship between drug use and mental health. The present study aims to provide the necessary understanding for the improvement and development of treatment strategies for mental health conditions.

Previous research has mainly focused on the specific effects of certain drugs of abuse on their own, yet drugs are often combined together concurrently and simultaneously (Roche et al., 2019); this is often due to common sociocultural influences (Bobo and Husten, 2000). Concurrent drug use is thought to reduce treatment efficacy by increasing drug cravings and drug intake, and has been linked with greater risk of mental health problems (McKee and Weinberger, 2013; Subbaraman and Kerr, 2015; Roche et al., 2019). Importantly, the relationship between drug consumption and mental health is not a simple causational one (NIDA, 2021). Risk factors may render some people more vulnerable to the effect of drugs. Of course, there are many types of risk factor which can influence this relationship, mainly those that are associated with the individual, such as family history, or environmental factors, such as poor socioeconomic status or a stressful environment (NIDA, 2021). One factor that falls in-between these factors is a person’s chronotype, which influences their daily activity patterns. The pattern of daily activity levels is determined by an individual’s circadian clock. The calibration of these circadian clocks vary from person to person, with a wide range of patterns throughout the population. Those with earlier behavioral patterns are termed early chronotypes and their later counterparts being late chronotypes, colloquially known as “early larks” and “night owls,” respectively (Roenneberg et al., 2003). The factors that determine an individual’s chronotype can be intrinsic, such as genetic influences or age, but these influences are not fixed. Chronotype can also change, even on a daily basis, in response to extrinsic factors (“Zeitgebers”) such as light exposure or working patterns (Roenneberg et al., 2004). Changes in working patterns and light exposure may have been influential in many peoples’ shift to a later chronotype during the pandemic.

Late chronotypes have been associated with impaired emotional and reward processing, which may impact on their wellbeing, specifically on their susceptibility to developing anxiety and depression (Díaz-Morales, 2015; Merikanto et al., 2015; Antypa et al., 2016; Taylor and Hasler, 2018; Cox and Olatunji, 2019). As drugs of abuse also influence the same systems by producing a pleasurable buzz or relaxation, it is not surprising that individuals with altered emotional and reward processing, as seen in late chronotypes, are more likely to use drugs (Patterson et al., 2016; Hasler et al., 2017a,b; Hug et al., 2019). Both factors, chronotype and drug consumption, are likely to be linked to alteration of reward processing, and it has been hypothesized that there may be a relationship between them, i.e., they may have a combined effect on the same systems and may cause an even greater impact on mental health. In fact, chronotype seems to be an under-recognized link between drug use and mental health (Hasler et al., 2017a). The present study aims to elucidate the effects of chronotype on mental health and the influence of drug consumption on this relationship, using data collected before the pandemic. We hypothesize that the increased use of substances such as tobacco, cannabis and alcohol in late chronotypes will mediate the relationship between late chronotype and poorer mental health.

## Materials and Methods

### Study Sample

The protocol was approved by the Cambridge Psychology Ethics Committee. A total of 449 individuals participated in the study using the online Qualtrics survey platform.^1^ The study was advertised by posting on websites and printed adverts in Cambridge (United Kingdom) and the surrounding regional area. After exclusion due to incomplete responses or invalid responses, two hundred and nine participants (50% male) with a mean age of 25.4 (± 8.4 standard deviation, SD) were included in the final sample. The age range and gender distribution were not significantly different in included and excluded groups. The identities of the participants remained anonymous, who were not paid for their contribution. This method of online data collection has been validated in previous studies (Ersche et al., 2017, 2019; Strickland and Stoops, 2019).

### Study Measures

All participants provided information about their demographics and completed five questionnaires to assess chronotype, alcohol and cannabis consumption, tobacco use, and mental health status.

#### Chronotype

The Munich Chronotype Questionnaire (MCTQ) is one of the most widely used measures to assess individual variation of the chronotype and has been validated with other chronotype measures (Roenneberg et al., 2003; Santisteban et al., 2018). The MCTQ contains 19 questions examining wake and sleep timing (for both work and free days). This continuous measure is unique in its ability to evaluate chronotype by using the midpoint between sleep onset and offset on free days (Wittmann et al., 2010). Midsleep on free days (MSF) is used as a measure for chronotype, which should be corrected due to potential sleep debt that has been incurred on weekdays. Sleep debt on week days leads to sleep recovery on free days, which increases sleep duration on free days (SD_f_), which would confound the measure of MSF. Weekend sleep recovery has thus been suggested to serve as a protective factor, reflecting the individual’s ability to catch up on lost sleep over the weekend. Weekend sleep recovery is calculated using the formula: weekend recovery (WR) = SD_f_- SD_week_, and MSF is corrected by using the difference between SD_f_ and average sleep duration (SD_week_). Sleep-corrected MSF (MSF_*sc*_) is then calculated using the formula: MSF_*sc*_ = MSF – (SD_f_ – SD_week_)/2. In the present study, we used MSF_sc_ as our final measure for chronotype.

#### Alcohol Consumption

Was assessed using the Alcohol Use Disorders Identification Test (AUDIT), recommended by the World Health Organization to measure harmful alcohol use (Saunders et al., 1993). The AUDIT contains ten items covering alcohol consumption, drinking behaviors and alcohol-related problems. Assessment of both alcohol consumption and drinking patterns can differentiate between binging and daily behaviors. Each item is scored 0 to 4, giving a maximum score of 40. Higher scores indicate more harmful use of alcohol, and a score of 8 or higher can be used to diagnose hazardous or harmful alcohol consumption.

#### Cannabis Consumption

Was assessed using the Revised Cannabis Use Disorders Identification Test (CUDIT-R). This is an eight-item questionnaire based on the AUDIT developed to screen for problematic cannabis use (Adamson et al., 2010). Each item is scored 0 to 4, giving the questionnaire a maximum score of 32. The CUDIT-R assesses frequency of use, cannabis consumption and cannabis-related problems. Higher scores indicate more harmful use of cannabis, with a score of 9 or higher indicating cannabis use disorder (Marshall, 2013).

#### Tobacco Use

Was as evaluated using the Fagerström Test for Nicotine Dependence (FTND), which is a well-established instrument to assess the intensity of physical addiction to nicotine (Heatherton et al., 1991). It is a six-item questionnaire with a maximum score of 10; a higher score indicates more harmful cigarette consumption and, by extension, nicotine dependence. Scores of 6 and above are seen as an indicator of high dependence (Hall et al., 2018). The FTND mainly assesses cigarette consumption and timing of smoking habits.

#### Combined Drug Use

Tobacco, alcohol and cannabis use scores were summed together to generate a measure of combined drug use, reflecting total drug burden on each participant. Scores from the FTND, AUDIT and CUDIT-R were summed together and then standardized to generate the combined drug use variable.

#### Mental Health

We have used the Depression, Anxiety and Stress Scale (DASS-21), which is a 21-item questionnaire to assess symptoms of depression, anxiety and stress (Lovibond and Lovibond, 1995). Each item has a statement that requires a rating with regard to frequency, with responses “Did not apply to me at all”, “Applied to me to some degree, or some of the time”, “Applied to me to a considerable degree, or a good part of the time” and “Applied to me very much, or most of the time” corresponding to 0, 1, 2, and 3 points, respectively. This questionnaire has three sections, with scales that distinguish well between depression, anxiety and stress-related pathologies. Each subscale has a maximum score of 21, and these scores can be summed to generate an overall mental health score. The three subscales for depression, anxiety and stress include items that measure dysphoric mood, such as sadness or worthlessness; physical arousal, panic attacks and fear as well as tension and irritability and overreaction to stressful scenarios, respectively. On each subscale, higher scores indicate higher severity of depression, anxiety or stress, and are summed up to a total score. The DASS-21 can be used to identify subclinical levels of these conditions, with established cut-off values for mild, moderate, severe and extremely severe depression, anxiety or stress (Lovibond and Lovibond, 1995). The detection of subclinical symptoms will allow us to more precisely determine the mechanism of chronotype’s effect on mental health.

### Statistical Analysis

First, we created multivariate linear regression models between chronotype (MSF_*sc*_) and total DASS-21 score, depression, anxiety and stress subscales. Chronotype was used as the dependent variable and the DASS-21 scores were the independent variables. As chronotype has a strong link to age, with changes in chronotype being observed from childhood to adolescence to adulthood (Roenneberg et al., 2004), age was included as a co-variate. We also included education level, which is often related to drug use and should be controlled for in the analysis (Crum et al., 1993; Fergusson et al., 2003; Tomioka et al., 2020). Our sample was gender balanced, with 50% male and female participants, but we decided not to explore the effects of gender as the link between gender and chorotype has been questioned (Randler and Engelke, 2019). To ensure that our findings are not confounded by gender, we ran a separate analysis using gender as a covariate (see also Supplementary Material).

Significant multivariate associations between chronotype and mental health scores were taken forward to mediation analysis. This is a statistical technique that determines whether the variance in the relationship between a dependent variable, X, and an independent variable, Y, can be explained by a mediating variable, M (Imai and Yamamoto, 2013). Statistical analyses were completed using R 3.6.1 and the “mediation” package 4.4.7.17 (Imai and Yamamoto, 2013; R Core Team, 2019). All tests were two-tailed with α = 0.05. We deliberately used the same method of analysis as previously described by Wittmann et al. (2010) to make our findings comparable with prior work.

## Results

### Sample Characteristics

The study sample was representative of the national average with regard to ethnicity (76% white), native language (85% native English speaking) and employment (6% unemployed). The education levels were above the national average as 70% have attended/are attending higher education. The majority of participants (81%) subjectively related their health status as either excellent or good, whereas only 0.9% considered their health to be rather poor. The sample had a large proportion of harmful alcohol (49%) use and harmful cannabis use (39%), with comparatively lower high dependence smoking (3%). It is of note that female participants reported significantly poorer mental health compared with their male counterparts (DASS-21 total; *t* = 2.77, *p* = 0.006), which was driven by significantly higher scores on both the anxiety subscale (*t* = 2.85, *p* = 0.005) and the stress subscale (*t* = 2.91, *p* = 0.004). By contrast, male participants reported significantly higher levels of nicotine (FTND; *t* = 2.84, *p* = 0.005) and cannabis use (CUDIT-R; *t* = 3.21, *p* = 0.002) compared with their female peers.

### Relationships Between Chronotype and Mental Health Measures

Table 1 shows the results of the multivariate regression analyses. Chronotype (MCTQ) was associated with total DASS-21 score (β = 0.16, *p* = 0.022) and anxiety (β = 0.18, *p* = 0.009). MCTQ was not statistically associated with depression or stress. During each regression analysis, both variables age and education were used as covariates. Importantly, the significant associations did not change when gender was used as an additional covariate (see Supplementary Table 1). Weekend sleep recovery was not correlated with DASS-21 total score (*p* = 0.986) or any of the subscales [DASS-21 anxiety (*p* = 0.688), DASS-21 depression (*p* = 0.700) or DASS-21 stress (*p* = 0.884)] suggesting that increased sleep over the weekend was unrelated to participants’ mental health. Consequently, weekend sleep recovery was not used in any further analyses. Combined drug use was used as a mediating variable to further investigate the multivariate relationships that were found.

**TABLE 1:** Multivariate associations between chronotype (as assessed by the MCTQ) and mental health (as assessed by the DASS-21).

	DASS-21 Total score	DASS-21 Anxiety	DASS-21 Depression	DASS-21 Stress
Chronotype	**0.16 (0.022)**	**0.18 (0.009)**	0.12 (0.10)	0.12 (0.082)
Age	**−0.15 (0.032)**	**−0.15 (0.023)**	−0.07 (0.32)	**−0.17 (0.015)**
Education	**−0.23 (<0.001)**	**−0.26 (<0.001)**	**−0.16 (0.018)**	**−0.20 (0.003)**

*Bold values are significant at p < 0.05.*

**FIGURE 1:**
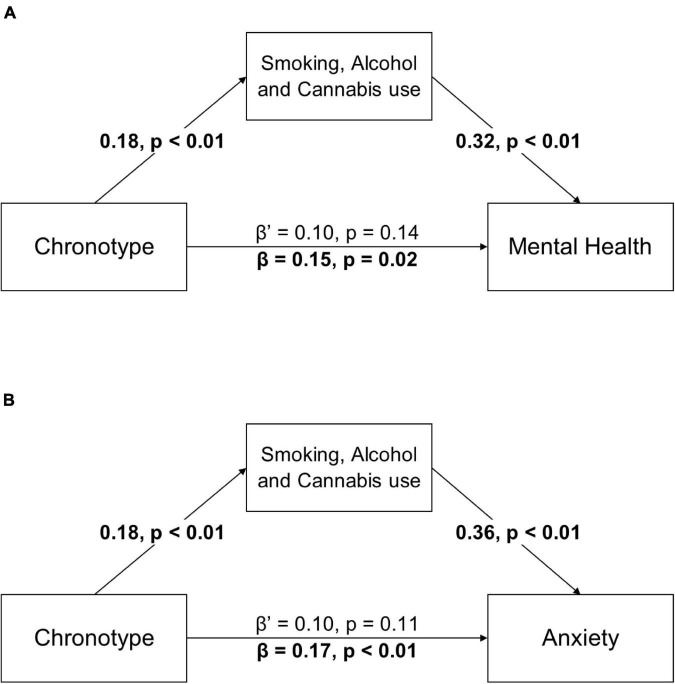
Combined variable of smoking, alcohol and cannabis use fully mediates the relationship between chronotype and mental health score **(A)** and the relationship between chronotype and anxiety **(B)**. β is the coefficient before mediation and β’ is the coefficient after mediation. All β coefficients are standardized. Age and education were used as covariates in this model but were not included in the figure. Significant paths are highlighted in bold.

The relationship between chronotype and total DASS-21 score (β = 0.15, *p* = 0.024) became non-significant when smoking, alcohol use and cannabis use were controlled for (β’ = 0.10, *p* = 0.138), showing a full mediation effect. When tobacco, alcohol and cannabis use were used as a combined mediator, the relationship between chronotype and anxiety disappeared (β = 0.17, *p* < 0.01; β’ = 0.10, *p* = 0.11) (see Figure 1). This full mediation effect was also present when gender was used as an additional covariate in the analyses (see Supplementary Figure 1).

## Discussion

In light of the steady increase in drug use and mental health problems worldwide (Twenge et al., 2019; UNODC, 2021), we aimed to elucidate the relationship between these phenomena and the possible influence of individual variation in chronotype on this relationship. As predicted, we identified multivariate associations between chronotype and mental health more generally, and with anxiety in particular. Late, or evening, chronotype was associated with higher anxiety and poorer overall mental health, suggesting that higher anxiety levels experienced by late chronotypes may have driven their significantly poorer overall mental health status. Whether or not participants had the chance to catch up on sleep over the weekend (weekend sleep recovery) did not seem to affect their mental health, suggesting that it had no protective effect. Specifically, the combined consumption of tobacco, alcohol and cannabis, which is highly prevalent not just in the United Kingdom (UNODC, 2021), fully mediated both of these relationships. These results suggest that the association between late chronotype and poorer mental health is driven by increased overall drug consumption in late chronotypes, rather than chronotype itself.

### Poor Mental Health in Late Chronotypes Is Influenced by Drug Use

Prior research suggests that the effects of chronotype on mental health are determined by tobacco smoking alongside alcohol use (Wittmann et al., 2010). Our work not only replicated these effects using different measures, but also showed that concomitant cannabis use, which is highly prevalent worldwide (Hall and Degenhardt, 2007; Hasler et al., 2017b; UNODC, 2021), has a strong influence on the relationship between chronotype and mental health. In other words, with the inclusion of cannabis in the model, variation in overall drug consumption has shown to be fully responsible for the relationship between chronotype and mental health. The increased combined use of drugs in late chronotypes, rather than the use of any particular drug, plays a causal role in their poorer mental health status. This combined drug use, either simultaneously or concurrently, also predicts worse mental health outcomes and treatment efficacy (Subbaraman and Kerr, 2015).

### Melatonin-Dopamine Interaction: A Putative Mechanism Underlying the Mediation

Although the influence of chronotype on mental health and drug use is known, the mechanism by which they interact is still elusive. A biological mechanism may explain such interactions. Both chronotype and drug use have been linked to alterations in reward processing (Hasler et al., 2017a; Taylor and Hasler, 2018), which are modulated by dopamine signaling (Arias-Carrión et al., 2010). Changes in dopamine transmission could thus mediate the relationships between the effects of the drugs of abuse, mental health and chronotype. Interestingly, late chronotype has also been associated with delayed melatonin release (Lack et al., 2009). Melatonin acts as a biochemical “signal of darkness” which entrains the sleep-wake and neuroendocrine cycles, even in the absence of environmental light changes (Zisapel, 2018). Consequently, melatonin has been linked with depression, as exemplified by the high prevalence of sleep and circadian changes in this condition (Tonon et al., 2021). The effects of melatonin are likely to extend beyond depression, as sleep changes are very common in many psychiatric disorders, and are often used as a diagnostic criterion (APA, 2013). The biochemical interaction between melatonin and dopamine may be responsible for this link in mental health disorders (Zisapel, 2001). It is therefore tempting to speculate that late chronotypes have disrupted signaling of both of these molecules, which may contribute to their increased susceptibility to the drug-related effects on mental health. Importantly, the effects of drug consumption on late chronotypes may be two-fold; evening types may not only use more drugs but they may also be more susceptible to the negative psychological effects of drugs.

Possibly, higher drug consumption in later chronotypes is due to the lack of synchrony between their biological alertness and their societal responsibilities (Wittmann et al., 2006). It is also of note that consumption patterns differ with stimulant and depressant drugs. For example, increased use of nicotine and caffeine may increase morning alertness in late chronotypes, whereas alcohol and possibly also cannabis, may decrease their high alertness at night time in order to sleep (Adan, 1994). Indeed, drug consumption is commonly used as a remedy for people with sleep problems, a common challenge for late chronotypes (Gulick and Gamsby, 2018). Interestingly, chronic use of alcohol, cannabis and tobacco have been reported to decrease rapid-eye-movement sleep duration, potentially leaving these late chronotypes even less rested, perpetuating their existing sleep problems and contributing to poorer mood and cognition (Yules et al., 1967; Stein and Friedmann, 2005; Bolla et al., 2008; Jaehne et al., 2009; Gulick and Gamsby, 2018).

Late chronotypes may also have increased sensitivity to the negative mental health effects of these drugs. Evening chronotypes have a 2–3 h delay in their circadian melatonin release and this delay is associated with higher negative emotion (Lack et al., 2009; Dolsen and Harvey, 2018). Melatonin has been shown to inhibit dopamine signaling, which may cause the increased presence of anxious or depressive symptoms in those with dysregulated melatonin (Zisapel, 2001; Zarrindast and Khakpai, 2015; Belujon and Grace, 2017). Drugs of abuse including alcohol, cannabis and tobacco dysregulate both dopamine and melatonin release, highlighting that overall drug consumption may exacerbate the existing signaling disruption in late chronotypes (Ursing et al., 2005; Koch et al., 2006; Rupp et al., 2007; Oleson and Cheer, 2012; Le Foll et al., 2014; Budygin and Weiner, 2015). Both late chronotype and high overall drug consumption could have an additive effect on dysregulating melatonin, which may in turn impact on dopamine-dependent reward signaling in the brain of late chronotypes to an even greater extent.

Existing mental health problems can also be a strong predictor of future drug abuse, with drug use and mental health having a bidirectional relationship (Swendsen et al., 2010). Both factors in this relationship can give rise to a negative spiral (Burdzovic Andreas et al., 2015). Late chronotypes may be more vulnerable to falling into this behavioral cycle *via* the proposed melatonin-dopamine interaction. However, further research is clearly warranted to support this assertion.

### Possible Implications for Treatment

Our study has highlighted that the association between late chronotype and poorer mental health is driven by increased anxious symptoms. Anxiety disorders are one of the most common psychiatric disorders, with a high burden of illness (Bandelow et al., 2017). Treatment for anxiety is necessary, yet many patients do not receive great benefit from treatment. Most clinical trials of treatments for anxiety disorders only report response rates of 50–60%, and remission rates of only 25–35% (Roy-Byrne, 2015). The identification of risk factors may allow us to recognize vulnerable individuals early and potentially stratify patients to define the most efficacious treatments for the various subdivisions within this heterogeneous anxious population.

Patients can be assessed for chronotype, allowing for intentional screening for anxious symptoms during consultations of late chronotypes that report harmful drug use. Local support groups and talking therapies can then be recommended to reduce drug use and anxious symptoms, respectively. With patient chronotype information, treatments can become more personalized; efficacy of treatments may change depending on chronotype (Oldham and Ciraulo, 2014). This understanding of the relationship between chronotype, mental health and drug use may also be influential in the management of current public mental health, with both drug use and late chronotype having increased since the beginning of the COVID-19 pandemic.

### Strengths, Weaknesses, and Future Outlook

The present study has several strengths in terms of measures and practical implications. The continuous measures of drug use (AUDIT, CUDIT-R, and FTND) assess not only presence, or absence, of drug use but the severity of drug use, which would be a more accurate indicator of the resultant mental health effects. Drug use was assessed in parallel as drugs are often used concurrently, which provides a more accurate representation of use patterns in the general public. We also measured participants’ mental health status using a continuous measure, i.e., the DASS-21 score, which is able to detect subclinical symptoms of depression, anxiety and stress as well as generating separate scores for each of these aspects of mental health. This allowed us to elucidate the relationship between chronotype and anxiety specifically. We also would like to point out the relatively large sample size of the present study, which was representative of the general population of East Anglia (United Kingdom).

Clearly, it would have been desirable to assess the relationships between chronotype, mental health and drug use longitudinally, as mental health and drug use have a bidirectional relationship. A future longitudinal study would be able to adequately investigate whether there is a causal relationship between increased drug use and poorer mental health in late chronotypes. For our study, it was not possible to adequately substantiate this claim as baseline mental health prior to drug use was not recorded. Clearly, the findings of this study may be more speculative than results obtained by longitudinal research. However, we have discussed the most likely interpretations of these data but also acknowledge that there may be even more. Moreover, the timing of drug use has not been recorded in this study, which would have been informative regarding the daily motivations for drug consumption, which could aid in the design of future therapeutic interventions. The age of our sample was relatively young (mean = 25 years), most likely due to the method of data collection being an online survey. Although the elderly are less likely to consume drugs, especially cannabis, the relationships shown in this study are currently limited to younger adults. Future studies may want to consider using multiple data collection methods to reach a wider age range, to allow our investigations to yield results that are generalizable amongst the wider population. However, these findings are still potent as the co-occurrence of mental health disorders and high levels of drug use are particularly prevalent in young people.

## Conclusion

We have shown that later chronotype is associated with poorer mental health, primarily through increased anxiety. These relationships are fully mediated by combined use of alcohol, tobacco and cannabis. This understanding suggests that monitoring and treatment, especially for anxiety, can be personalized using patient chronotype information in order to optimize the efficacy of future interventions. Late chronotypes with mental health disorders can be recommended counseling and mutual support groups to minimize their drug intake, which may act to relieve their symptoms.

## Data Availability Statement

The raw data supporting the conclusions of this article will be made available by the authors, without undue reservation.

## Ethics Statement

The studies involving human participants were reviewed and approved by the Cambridge Psychological Research Committee. The patients/participants provided their written informed consent to participate in this study.

## Author Contributions

KDE acquired the funding, designed the study and wrote the protocol. JF managed the literature searches, analysed the data and wrote the first draft of the manuscript. JS assisted with the statistical analysis and contributed to the interpretation of the data. All authors contributed to and have approved the final manuscript.

## Conflict of Interest

The authors declare that the research was conducted in the absence of any commercial or financial relationships that could be construed as a potential conflict of interest.

## Publisher’s Note

All claims expressed in this article are solely those of the authors and do not necessarily represent those of their affiliated organizations, or those of the publisher, the editors and the reviewers. Any product that may be evaluated in this article, or claim that may be made by its manufacturer, is not guaranteed or endorsed by the publisher.

## Footnotes

1
https://caret.eu.qualtrics.com
