# Supplementary material for: Drug Use in Night Owls May Increase the Risk for Mental Health Problems
Source: Front Neurosci. 2022 Jan 11;15:819566. doi: 10.3389/fnins.2021.819566 (PMC8787192; doi:10.3389/fnins.2021.819566)
Supplement: Supplementary file 1 [file Data_Sheet_1.pdf]

Supplemental Material

Drug use in night owls may increase the risk for mental health problems

J Fernando, J Stochl, KD Ersche✉

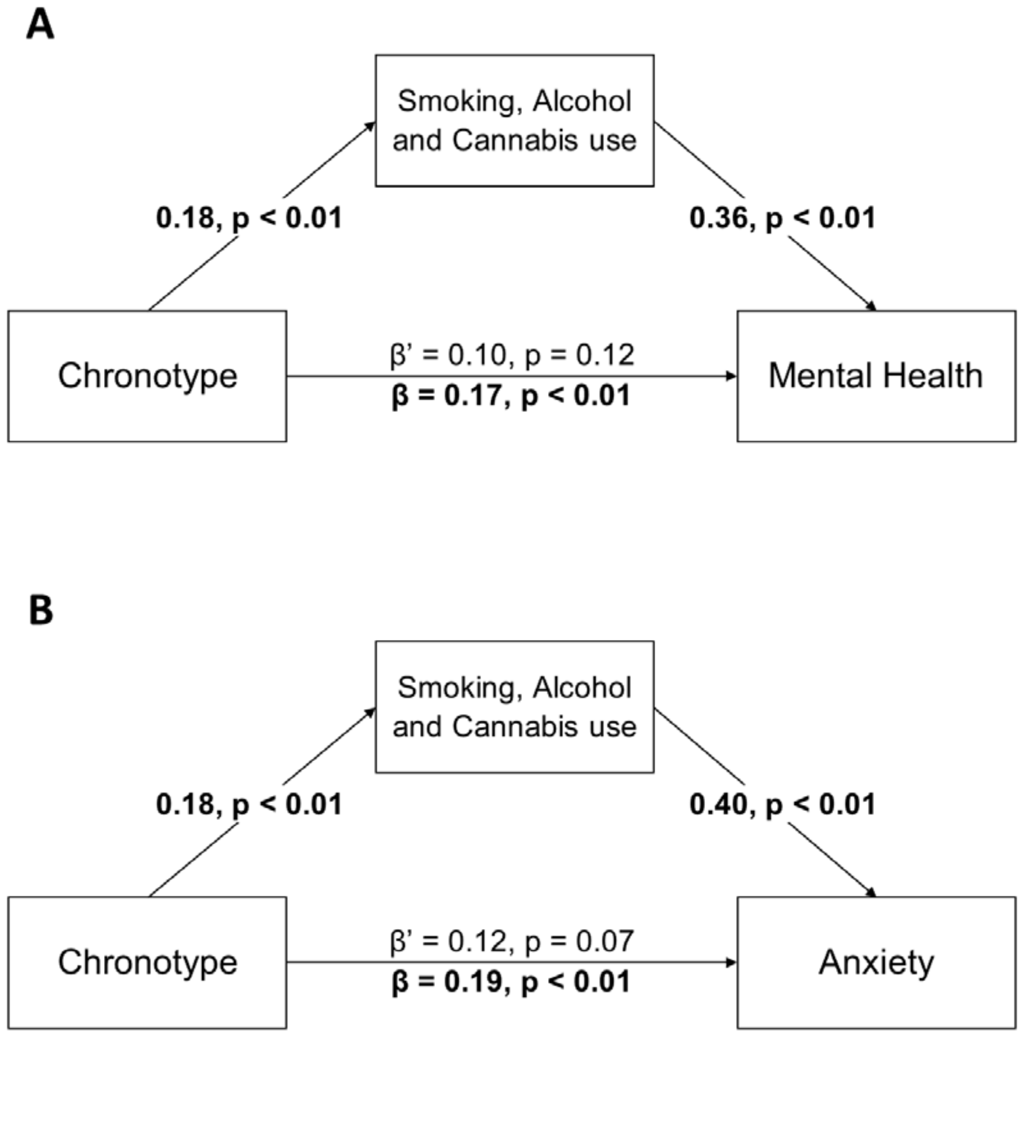

Figure S1. Combined variable of smoking, alcohol and cannabis use fully mediates the relationship between chronotype and mental health score **(A)** and the relationship between chronotype and anxiety **(B)**. β is the coefficient before mediation and β′ is the coefficient after mediation. All β coefficients are standardized. Age, gender and education were used as covariates in this model but were not included in the figure. Significant paths are highlighted in bold.

Table S1. Multivariate associations between chronotype (as assessed by the MCTQ) and mental health (as assessed by the DASS-21) with covariates of age, education and gender.

	DASS-21 Total Score	DASS-21 Anxiety	DASS-21 Depression	DASS-21 Stress
Chronotype	**0.17 (0.013)**	**0.19 (0.003)**	0.12 (0.080)	0.13 (0.054)
Age	-0.11 (0.092)	**-0.12 (0.072)**	-0.05 (0.47)	**-0.13 (0.047)**
Education	**-0.25 (<0.001)**	**-0.27 (<0.001)**	**-0.17 (0.013)**	**-0.22 (0.001)**
Gender	**0.39 (0.003)**	**0.40 (0.002)**	**0.23 (0.085)**	**0.39 (0.003)**
